# Supplementary material for: Impacts of the global food system on terrestrial biodiversity from land use and climate change
Source: Nat Commun. 2024 Jul 9;15:5750. doi: 10.1038/s41467-024-49999-z (PMC11233703; doi:10.1038/s41467-024-49999-z)
Supplement: Supplementary file 3 — Description of Additional Supplementary Files [file 41467_2024_49999_MOESM3_ESM.pdf]

Description of Additional Supplementary Files

Supplementary Data 1. Characterisation factors for land-driven species richness change (species).

Supplementary Data 2. Characterisation factors for land-driven rarity-weighted richness change (range fractions).

Supplementary Data 3. Characterisation factors for GHG-driven species richness change (species). For the purposes of the analyses, the 19 GHG-driven factors listed were summed into a single characterization factor or a single factor for each gas (carbon dioxide, methane and nitrous oxide).

Supplementary Data 4. Characterisation factors for GHG-driven rarity-weighted richness change (range fractions). For the purposes of the analyses, the 19 GHG-driven factors listed were summed into a single characterization factor or a single factor for each gas (carbon dioxide, methane and nitrous oxide).

Supplementary Data 5. Areas of the EXIOBASE trade regions (km^2) as calculated from ESRI country shapefiles[15].

Supplementary Data 6. The 2011 populations of the EXIOBASE trade regions as estimated by CountryEconomy.com[16] (Taiwan) and the World Bank[17] (all other regions).

Supplementary Data 7. Production and consumption footprints of land use (km^2), land-driven species richness (species × km^2), land-driven rarity-weighted richness (range fractions × km^2), GHGdriven species richness (species × km^2) (total, CO2, CH4 and N2O) and GHG-driven rarity-weighted richness (range fractions × km^2) (total, CO2, CH4 and N2O) for all regions and all food-related products for the year 2011.

Supplementary Data 8. Production footprints of land use (km^2), land-driven species richness (species × km^2), land-driven rarity-weighted richness (range fractions × km^2), GHG-driven species richness (species × km^2) (total, CO2, CH4 and N2O) and GHG-driven rarity-weighted richness (range fractions × km^2) (total, CO2, CH4 and N2O) for aggregated world regions and all food-related products for the year 2011.

Supplementary Data 9. Production per km2 footprints of land use (km²), land-driven species richness (species x km²), land-driven rarity-weighted richness (range fractions x km²), GHG-driven species richness (species x km²) (total, CO2, CH4 and N2O) and GHG-driven rarity-weighted richness (range fractions x km²) (total, CO2, CH4 and N2O) for world regions and all food-related products for the year 2011.

Supplementary Data 10. Consumption per capita footprints of land use (km²), land-driven species richness (species x km²), land-driven rarity-weighted richness (range fractions x km²), GHG-driven species richness (species x km²) (total, CO2, CH4 and N2O) and GHG-driven rarity-weighted richness (range fractions x km²) (total, CO2, CH4 and N2O) for world regions and all food-related products for the year 2011.

References:

[15] ESRI (2015). Countries WGS84, https://hub.arcgis.com/datasets/a21fdb46d23e4ef896f31475217cbb08_1/data, accessed 11 November 2021

[16] CountryEconomy.com. https://countryeconomy.com/demography/population/taiwan?year=2011, accessed 26 October 2021.

[17] World Bank (2021). Data Bank, https://databank.worldbank.org, accessed 26 October 2021.
